# Update on the association between Helicobacter pylori infection and asthma in terms of microbiota and immunity

**DOI:** 10.1186/s13223-024-00870-2

**Published:** 2024-01-14

**Authors:** Mengmeng Liu, Yong Wang, Bing Du

**Affiliations:** Department of Gastroenterology and Hepatology, Second Affiliated Hospital of Harbin Medical University, Harbin, China

**Keywords:** Helicobacter pylori, Allergic diseases, Asthma, Hygiene hypothesis, Gut microbiota, Tolerogenic dendritic cells, Helper T cells

## Abstract

H. pylori is a gram-negative bacterium that is usually acquired in childhood and can persistently colonize the gastric mucosa of humans, affecting approximately half of the world’s population. In recent years, the prevalence of H. pylori infection has steadily reduced while the risk of allergic diseases has steadily climbed. As a result, epidemiological research indicates a strong negative association between the two. Moreover, numerous experimental studies have demonstrated that eradicating H. pylori increases the risk of allergic diseases. Hence, it is hypothesized that H. pylori infection may act as a safeguard against allergic diseases. The hygiene hypothesis, alterations in gut microbiota, the development of tolerogenic dendritic cells, and helper T cells could all be involved in H. pylori’s ability to protect against asthma. Furthermore, Studies on mice models have indicated that H. pylori and its extracts are crucial in the management of asthma. We reviewed the in-depth studies on the most recent developments in the relationship between H. pylori infection and allergic diseases, and we discussed potential mechanisms of the infection’s protective effect on asthma in terms of microbiota and immunity. We also investigated the prospect of the application of H. pylori and its related components in asthma, so as to provide a new perspective for the prevention or treatment of allergic diseases.

## Introduction

The human gastrointestinal tract is home to billions of microorganisms that interact symbiotically with their hosts and play a critical role in both health and illness. H. pylori, a gastrointestinal microorganism, is one of the most studied bacteria. The network of interactions that H. pylori have constituted with its host is closely linked to all systems of the organism [1]. Numerous systemic illnesses, including neural, hematological, cardiovascular, dermatological, and allergic diseases are linked to H. pylori [2, 3]. Among them, the relationship between H. pylori infection and the risk of allergic diseases is becoming better known and is of some concern to the general public. The interaction of the human immune system and environmental factors leads to allergic diseases, and given the substantial regional heterogeneity of these diseases, it is likely that environmental factors play a significant role in their etiology [4]. As a result, growing evidence from research demonstrating an association between early H. pylori exposure and allergic diseases suggests that early life exposure to H. pylori may act as a preventative factor in the development of allergic disease [5, 6]. However, only a small number of studies have described the immune response to H. pylori and the relationship between the bacteria and the gut microbiota. This paper explored the relationship between H. pylori infection and asthma in terms of immunity and gut microbiota, as well as the use of H. pylori and its related components in the treatment of asthma. It also introduced the most recent developments in the correlation between H. pylori infection and allergic diseases.

## Association between H. Pylori infection and the risk of allergic diseases

### Association between H. pylori and asthma

Asthma is a heterogeneous disease with chronic airway inflammation, bronchial hyperresponsiveness and airway remodeling, and its pathogenesis is very complex [7]. In recent years there have been many studies on the association of H. pylori infection with the risk of asthma. Epidemiological studies have shown a decline in the prevalence of H. pylori infection in the Western World and in some developing countries in contrast to an increase in the incidence of asthma and allergic diseases [8]. Studies have demonstrated that H. pylori infection can prevent asthma [9, 10], and it has been noted that CagA-positive H. pylori infection is significantly negatively associated with the risk of asthma [11, 12] and may even be negatively associated with the severity of asthma [11]. A meta-analysis of 18 cross-sectional studies found that H. pylori infection, especially CagA-positive H. pylori infection, was inversely associated with the prevalence of asthma [13]. Another meta-analysis of 24 studies (8 case-control studies and 16 cross-sectional studies) reached the same conclusion [12]. However, there are questions about the negative association between H. pylori infection and the risk of asthma. Several studies suggest no correlation between H. pylori infection and asthma risk and do not support the notion that H. pylori infection has a protective effect against asthma [14–16]. The aforementioned study analyzed the correlation between H. pylori IgG antibody positivity and the incidence of asthma. A positive H. pylori IgG antibody indicates a previous H. pylori infection but does not necessarily imply a current infection. Therefore, we believe that further studies and experiments are necessary to support and confirm this discovery. Research by Wang et al. pointed out that H. pylori infection was significantly associated with a 1.38-fold increased risk of asthma. This indicates that the risk of asthma is significantly higher in patients with H. pylori infection than in subjects without H. pylori infection [17]. However, the methods of detecting H. pylori and possible H. pylori treatment during the follow-up were not fully addressed. Socioeconomic factors, as potential confounding factors, had not been taken into account in the study. We noticed that a relevant article raised doubts about the conclusion of the study [18]. Although the findings are slightly controversial to some extent, the negative association of H. pylori infection with asthma risk is supported by most scholars.

### Association between H. pylori and eosinophilic esophagitis

Eosinophilic esophagitis (EoE) is a chronic, immune-mediated inflammatory disease whose pathogenesis is not fully understood. The histology is characterized by eosinophil-dominated inflammation with clinical symptoms associated with esophageal dysfunction [19, 20]. Emerging evidence suggests that modifiable host factors and environmental allergen exposure may play a key role in the pathogenesis of eosinophilic esophagitis [21]. The gradual increase in the incidence of eosinophilic esophagitis and the decrease in the rate of H. pylori infection in recent years have given rise to speculation and discussion about the relationship between the two. A strong negative correlation between the presence of H. pylori and esophageal eosinophilia has been demonstrated [22]. The results of case-control studies and meta-analyses suggest that H. pylori infection is associated with a reduced risk of eosinophilic esophagitis [23, 24], but the protective effect of H. pylori infection against eosinophilic esophagitis has also been questioned as an uncritical claim that requires the exclusion of confounding factors associated with it and the demonstration of a causal rather than a coincidental trend relationship [25, 26].

### Association between H. pylori and food allergies or allergic rhinitis

The area of the relationship between H. pylori infection and allergic rhinitis has rarely been learned. A study in Japan indicated a negative correlation between H. pylori infection and the incidence of allergic rhinitis in young people [27]. However, there is no further evidence to support this conclusion. Similarly, there has been limited discussion about the relationship between H. pylori infection and food allergies. A systematic review described the relationship between them but did not come to a conclusive result [28]. However, subsequent studies have shown that H. pylori infection has a protective effect against food allergies, including ovalbumin allergy and peanut allergy [29, 30]. Further researches are needed to fully understand the mechanisms behind this relationship and to determine if H. pylori infection could potentially be used as a treatment or preventative measure for food allergies or allergic rhinitis.

## Mechanism of H. pylori protection against asthma

**Fig. 1:**
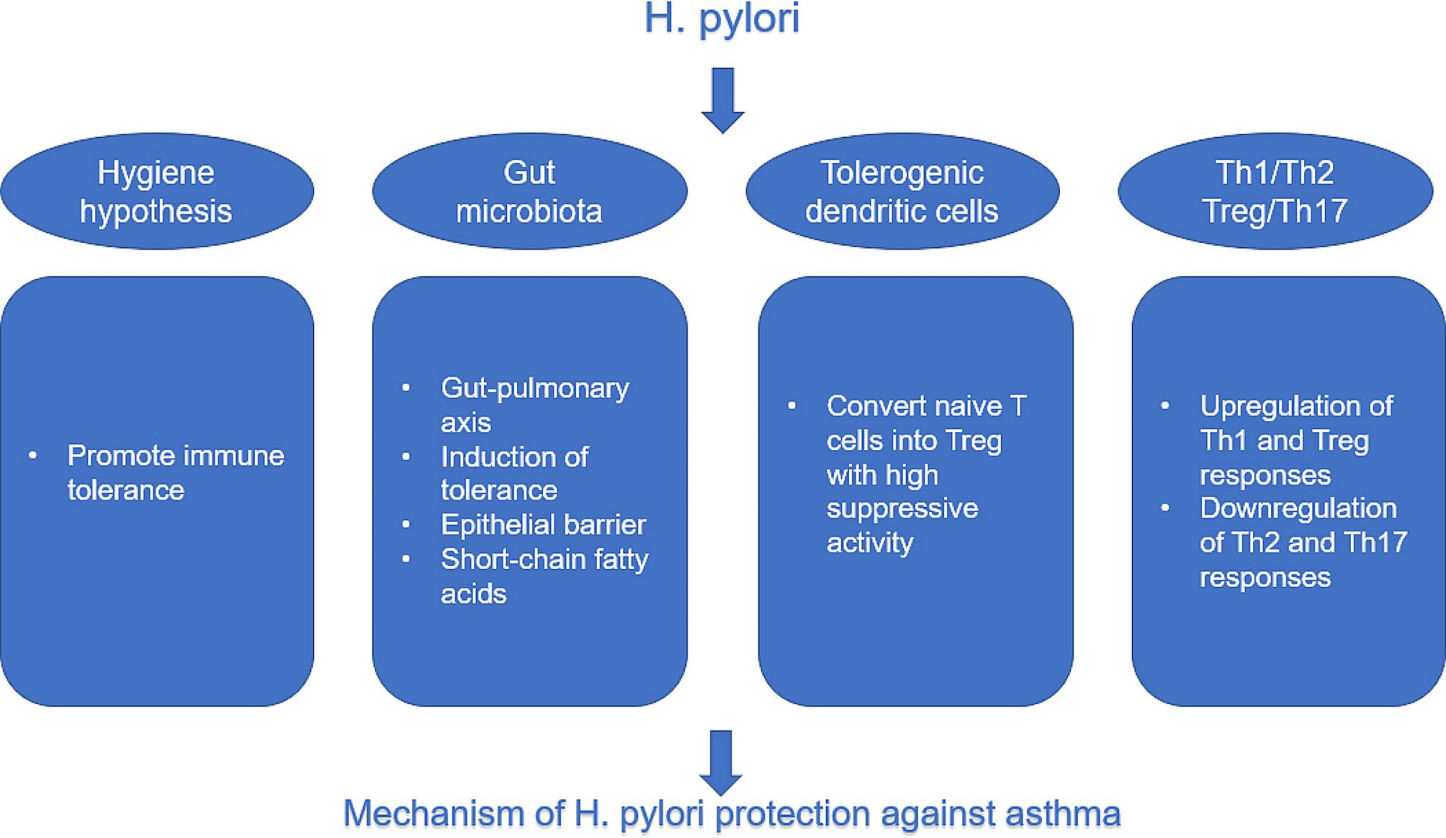
Pathways by which H. pylori exerts a protective effect against asthma

Genetics and environment are two factors essential for the development of asthma in patients. Genetics determines the patients’ special allergies, and susceptibility to asthma, and whether such patients develop the disease or not is highly related to environmental factors. H. pylori infection showed a significant negative association with asthma risk, but as an environmental factor, the specific pathophysiological mechanism by which it exerts a protective effect on asthma remains unclear. From the analysis of some previously published articles on the subject, it is hypothesized that H. pylori may exert its protective effect against asthma through several pathways(Fig. 1).

### Application of hygiene hypothesis to the protective effect of H. pylori on asthma

The “hygiene hypothesis,” which has been adopted by the infectious and chronic disease research community since the early 1990s, proposes that exposure to certain infectious agents may prevent the development of allergic diseases [12]. Poor hygiene and lower socioeconomic status increase the risk of exposure to bacteria or other antigens, and therefore to H. pylori infection [2, 31]. In recent years, with the improvement of people’s quality of life, hygiene conditions, and socioeconomic status, the rate of H. pylori infection has gradually decreased and the low prevalence of H. pylori infection could explain the recent high prevalence of allergic diseases [28]. Lack of exposure to infection early in life leads to defective immune tolerance, which in turn leads to increased susceptibility to allergic diseases such as asthma [21, 32], leading to the hypothesis that H. pylori infection exerts a protective effect against allergic diseases such as asthma by promoting immune tolerance. It was pointed out that the hygiene hypothesis can explain the negative correlation between H. pylori infection and allergic diseases. However, it only fits to IgE-mediated allergic diseases and not to non-IgE-mediated allergic diseases [33]. IgE-mediated allergic diseases are caused by immunoglobulin E (IgE)-mediated allergic reactions and are the most common type of allergy. Non-IgE-mediated allergic diseases are mediated by other immune cells, and the pathogenesis is very complex, but the incidence is low. Asthma is an IgE-mediated allergic disease, so it can be concluded that the hygiene hypothesis may explain the negative association between H. pylori infection and asthma.

### Alterations in the gut microbiota

The composition of the gut microbiota may regulate the onset and development of H. pylori-associated diseases. The composition of the gut microbiota influences the immune regulation of the body, and microbial drivers have significant effects on immune development, asthma susceptibility, and asthma pathogenesis [34]. It is known that H. pylori is strictly colonized within the human gastric mucosa and that H. pylori in the stomach may be affecting the intestinal microbiota in the following ways. Theoretically, H. pylori in the stomach can affect the intestinal microbiota by interacting with the body’s immune system and also by altering the local gastric environment. Alterations in the local gastric environment include reduced gastric acid and hypergastrinemia during H. pylori infection, with the low gastric acid environment promoting the entry of acid-sensitive bacteria into the distal intestine as probably the most important pathway of effect, leading to alterations in the composition and abundance of the gut microbiota [1]. Even perinatal H. pylori exposure can have a significant impact on the composition and diversity of the neonatal gastrointestinal microbiota [35]. Accordingly, it can be concluded that H. pylori infection affects the composition and abundance of the gut microbiota.

Ecological dysregulation caused by alterations in the composition and abundance of the gut microbiota plays a role in asthma [36, 37], especially in the development and progression of asthma in children [38–40]. The gut microbiota exerts its influence on asthma through several known pathways. The gut-pulmonary axis is an important link between the gut microbiota and the respiratory tract [36], and the metabolites produced by the gut microbiota may have an impact on the development of asthma through the gut-pulmonary axis pathway [41, 42]. The gut microbiota is a key regulator of the intestinal epithelial barrier and the immune response [43], which can act on asthma through the induction of tolerance and allergen penetration through the epithelial barrier [44]. In addition, short-chain fatty acids (SCFA) produced by dietary fiber metabolism by the gut microbiota can prevent asthma by affecting the host G protein-coupled receptor GPR 41, shaping pulmonary immune cell differentiation, and improving allergic airway inflammation [45].

Studies on the relationship between gut microbiota and asthma development in mothers and infants have shown that alterations in maternal gut microbiota composition affect the risk of asthma in infants [46]. Based on the conclusion that gut microecological dysbiosis has an impact on the development of asthma, it can be hypothesized that the gut microbiota could be a target for the treatment of asthma by altering its composition and abundance and thus exerting a therapeutic effect on asthma. Clinically used probiotics can have a preventive or therapeutic effect on asthma by regulating the gut microbiota [47, 48]. Some studies have also shown that alterations in the composition and abundance of the gut microbiota are not associated with the development of asthma. In a mouse experiment, the gut microbiota was found to be independent of reflecting airway hyperresponsiveness penh values [49]. In a cohort study of adults, no significant differences were found in the composition of the fecal microbiota between asthmatic and non-asthmatic patients [39]. The reasons for these results may be due to the underrepresentation of the fecal microbiota to the gut microbiota, the adult immune system is well developed and alterations in the gut microbiota do not or only slightly affect the adult immune system.

**Fig. 2:**
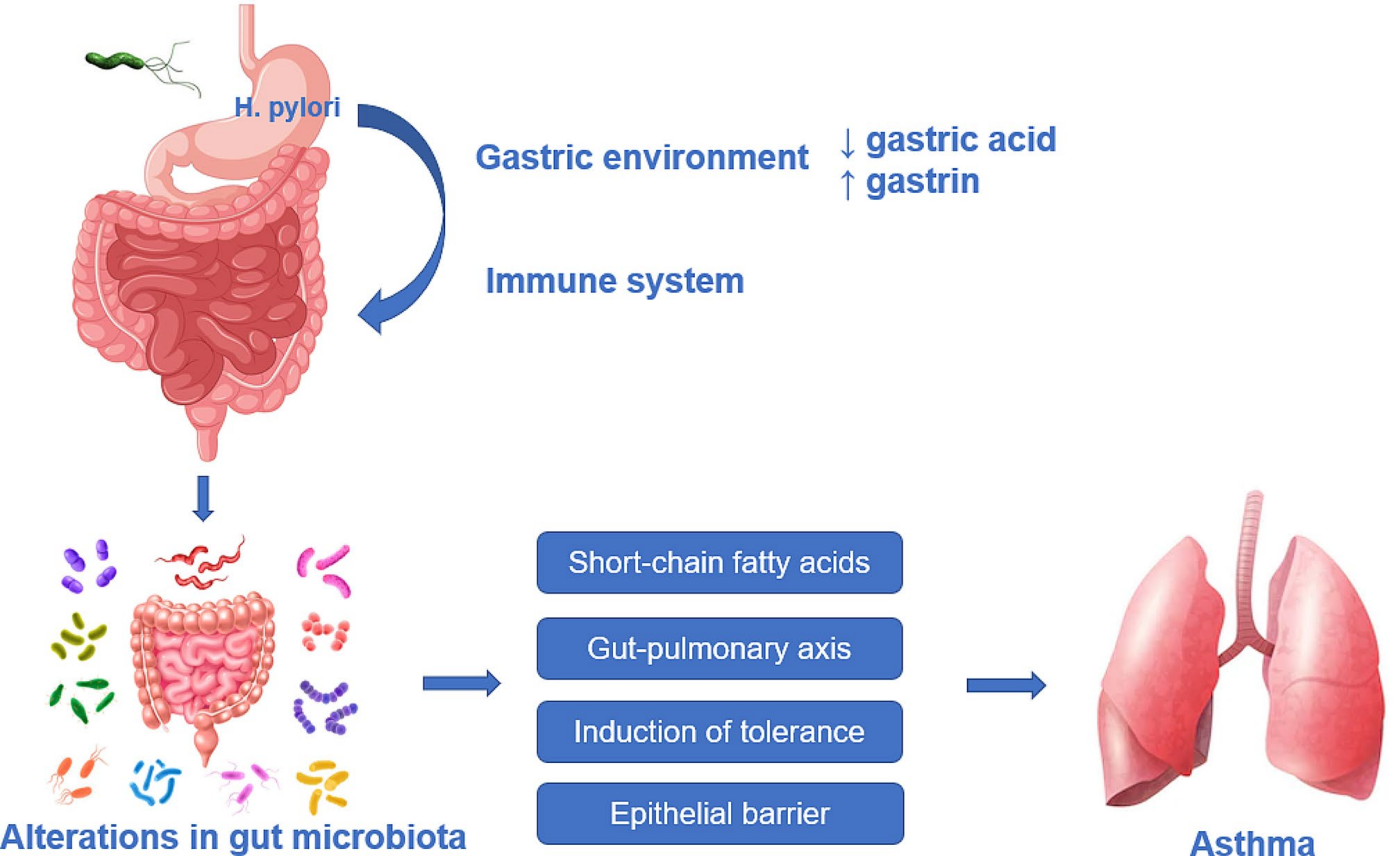
The pathways by which H. pylori affects the gut microbiota and the mechanisms by which the altered gut microbiota affects asthma

H. pylori infection can affect the composition and abundance of the gut microbiota through interactions with the body’s immune system and changes in the local gastric environment. The gut microbiota uses the gut-pulmonary axis as an important linkage pathway to exert a protective effect against asthma, either through metabolites or by modulating immunity(Fig. 2). However, a limitation of this research area is that in most of the relevant studies, the fecal microbiota is used instead of the gut microbiota, ignoring the microorganisms remaining in the gut, which may cause bias in the results. In the H. pylori-gut microbiota-asthma liaison pathway, ignoring the possible bias, the gut microbiota can serve as an emerging target for the prevention and treatment of asthma. Modification of the gut microbiota by certain drugs or treatments, which in turn exerts a protective effect against asthma.

### The critical role of tolerogenic dendritic cells in the protection of asthma by H. pylori

H. pylori inhibits lipopolysaccharide-induced dendritic cell (DC) maturation and is able to recode dendritic cells into tolerogenic dendritic cells [50, 51]. Some findings show that tolerant dendritic cells do not induce effector functions of T cells, but rather convert naive T cells into FoxP3 + Treg with high suppressive activity. FoxP3 + Treg can prevent airway inflammation and hyperresponsiveness, thus exerting a protective effect against asthma [50]. H. pylori can produce urease, which activates NLRP3, a component of cytoplasmic inflammatory vesicles, and stimulates the TLR2/ NLRP3/IL-18 axis [52]. IL-18 on this axis is a key cytokine for Treg to perform its function, IL-18 produced by dendritic cells is not only the basis for the conversion of CD4 + T cells into Treg but also for Treg to perform its function [2]. γ-glutamyl transpeptidase (GGT) and vacuolar cytotoxin (VacA) are virulence factors of H. pylori, and it was demonstrated that isogenic H. pylori mutants lacking GGT or VacA cannot prevent LPS-induced dendritic cells maturation or drive dendritic cells tolerance, thus the above two virulence factors play a key role in dendritic cell tolerance [53]. Based on the promoting effect of tolerogenic dendritic cells on Treg formation and the protective effect of Treg in asthma, it can be inferred that transforming sufficient numbers of dendritic cells into tolerogenic dendritic cells and maintaining their tolerance status is key for H. pylori to exert a protective effect against asthma.

### The immune balance of Th1/Th2 and Treg/Th17 cells

A large number of cells such as eosinophils, neutrophils, mast cells, and T lymphocytes are involved in the airway inflammation of asthma [54]. Among them, CD4 + T cells are the main lymphocytes that infiltrate the airways and play a crucial role in controlling asthma-related inflammation. Naive CD4 + T cells can differentiate into Th1, Th2, Th17, and Treg. Th1 cells produce IFN-γ, while Th2 cells produce IL-4, IL-5, and IL- 13 [55]. Th2-biased immune responses in genetically susceptible individuals may cause allergic diseases such as asthma [56]. It has been claimed that H. pylori infection affects the Th1/Th2 balance by influencing gastric hormones. When growth inhibitory hormone levels decrease and gastrin production increases, it suppresses the Th2 response and promotes the Th1 response [11]. The mechanism by which H. pylori prevents and protects against asthma may be to drive the Th1 inflammatory response and inhibit the Th2-mediated allergic asthmatic response [4, 5, 14]. In the clinic, upregulation of Th1 response or downregulation of Th2 response seems to be a target for the treatment of asthma, but it still needs to be explored and tested in the clinic. Treg and Th17 cells are functionally antagonistic to each other, and the balance of Treg and Th17 cells plays an important role in the development and progression of H. pylori and its associated diseases [57]. Excess IL- 17 has been found in sputum, bronchoalveolar lavage fluid (BALF), and lung tissue in chronic allergic airway inflammation [54]. It is hypothesized that both Th1/Th2 balance and Th17/Treg balance play a key role in the onset and persistence of asthma, and that asthma can be prevented and protected when Th1 and Treg are dominant in the ratio. One study experimented with the relationship between Th1 and Treg responses to H. pylori and allergen-specific IgE levels. The results showed a significant increase in IL- 10(+) Treg in the peripheral blood of H. pylori-infected individuals and correlated with a decrease in plasma IgE concentrations [58]. Th2 and its its cytokines are the basis of inflammation in asthma pathogenesis, and H. pylori exerts a protective effect against asthma by promoting the Th1 response and inhibiting the Th2 response. Th17 and its cytokines are also important in controlling asthma-associated inflammation, and Treg not only antagonism with Th17 but also directly suppresses airway inflammation and hyperresponsiveness in asthma. The protective effect on asthma that can be exerted by enhancing the Treg response is a currently available target for asthma treatment and is a very promising route for the treatment of asthma.

H. pylori affects the onset and development of asthma by influencing the balance of Th1/Th2 and Treg/Th17. This is one of the potential mechanisms, but it is still in the developmental stage, and the exact mechanism remains to be determined. Several factors influence the balance between Th1/Th2 and Treg/Th17. The Th1 response is mainly associated with autoimmune reactions, while the Th2 response is primarily linked to allergic reactions. Bacterial or viral infections can cause their imbalances, and H. pylori may be no exception. Further experiments are needed to explore the distinctiveness and dependability of this mechanism.

## Helicobacter pylori in the treatment of asthma

It has been shown that the protective effect of H. pylori infection against allergic airway disease does not require live bacteria and that treatment with H. pylori extracts is also effective in suppressing allergic airway disease [59]. Even perinatal exposure to H. pylori extract or its immunomodulator VacA can exert a protective effect against allergic airway disease, and this powerful protective effect occurs not only in the first but even in the second generation of offspring [35]. This shows the great scope for the development of H. pylori and its extracts in the prevention and treatment of allergic airway diseases such as asthma, and we may try to intervene in suspected asthma in newborns through perinatal exposure. Helicobacter pylori neutrophil-activating protein (Hp-NAP), the main virulence factor of H. pylori, is a modulator with anti-Th2 inflammatory activity for the prevention of IgE-mediated allergic reactions [60]. Hp-NAP is a member of an extensive superfamily of ferritin-like proteins, which are homopolymers of 12 tetrahelical bundle subunits containing iron ligands, and whose members mostly have DNA-protective functions under starvation conditions [60]. Hp-NAP plays an important role in the protection of H. pylori infection against allergic diseases and is one of the candidates for a new strategy of prevention and protection against allergic diseases. H. pylori neutrophil-activating protein was shown to prevent allergic asthma in mice. Experimental mice exposed to purified rNAP by intraperitoneal injection or inhalation showed a significant reduction of eosinophils in lung tissue and bronchoalveolar lavage fluid (BALF) after stimulated sensitization with nebulized ovalbumin (OVA), and also a significant reduction of inflammatory infiltration in lung tissue. In addition, the treatment group showed lower levels of IL-4 and IL- 13, higher levels of IL- 10 and IFN-γ, and lower levels of serum IgE compared to the control group [61]. Another similar study showed the same results, where a fusion protein CTB-NAP of cholera toxin B (CTB) and neutrophil-activating protein (NAP) was constructed on the surface of Bacillus subtilis, and oral administration of recombinant CTB-NAP spores was effective in preventing asthma in mice [60]. The prevention and treatment of asthma are systematic, the treatment of asthma focuses not only on the acute onset of symptoms but also on preventing the recurrence in clinical remission stage. Therefore, the above studies show the great potential of NAP in the prevention and treatment of allergic diseases such as asthma, but future experiments are still needed to verify whether NAP can cause side effects and toxic effects, and other adverse reactions in humans. Another substance, human protein S, enables a shift to Th1 through the Th1/Th2 balance and promotes Th1 cytokine secretion to exert a powerful protective effect on the development of allergic asthma [62]. It is clinically recognized that H. pylori eradication reduces the risk of gastric cancer, but based on its preventive and protective effects on allergic diseases such as asthma and other systemic diseases, the issue of H. pylori eradication should be considered with caution. Some studies have shown that eradication of H. pylori can restore the intestinal flora to a state similar to that of uninfected individuals [63–65], and others have shown that eradication treatment leads to short-term disruption of the intestinal flora, but that this disruption is restored within weeks to months [66–68]. The us e of H. pylori in the treatment of asthma opens the breadth of research on the association of H. pylori infection and asthma risk, with a novel perspective on the importance of H. pylori infection in asthma. However, the application of H. pylori and its extracts in the treatment of asthma still requires a large number of clinical trials to verify its safety and effectiveness and to exclude its possible adverse reactions.

## Conclusions

Many domestic and international scholars have made significant progress in recent years by conducting multi-dimensional and multi-angle discussions and studies on the relationship between extra gastric disorders and H. pylori. In terms of microbiota and immunity, this review summarizes recent developments in H. pylori infection and asthma. Topics covered include the relevance of H. pylori to allergic disease, potential mechanisms by which H. pylori infection exerts a protective effect on asthma, and the use of H. pylori in the treatment of asthma. According to the majority of studies, H. pylori infection has a strong negative correlation with the risk of a number of allergic disorders, including asthma and eosinophilic esophagitis.

The hygiene hypothesis suggests that exposure to certain infectious agents may prevent the development of allergic diseases such as asthma, and therefore it is hypothesized that H. pylori infection would exert a protective effect against asthma by promoting immune tolerance. Through a variety of mechanisms, H. pylori infection alters the composition and abundance of the gut microbiota, which in turn exerts a preventive and protective effect against asthma through the gut-pulmonary axis. Dendritic cells can be reprogrammed by H. pylori to become tolerogenic dendritic cells, and tolerogenic dendritic cells promote the production of Treg with high inhibitory activity. Both Th1/Th2 balance and Th17/Treg balance play a significant role in the onset and persistence of asthma and can prevent and protect against asthma when Th1 and Treg are dominant in the ratio. Many studies have demonstrated the great potential of H. pylori neutrophil-activating protein(NAP)in the prevention and treatment of allergic diseases such as asthma. H. pylori, its components, or extracts have certain preventive and therapeutic effects on asthma. It may represent a new way to treat asthma in the future, but it is not widely known by clinical staff. The eradication of H. pylori in asthmatic patients remains to be discussed.

There are still some unanswered questions despite the fact that the studies mentioned above showed an association between H. pylori infection and the risk of allergic disease. The detailed mechanisms that give rise to these correlations are not clear. The mechanisms may be closely interconnected. The hygiene hypothesis is a significant theory rooted in epidemiology. This hypothesis not only explains the negative correlation between H. pylori and asthma from an epidemiological perspective but may also account for other mechanisms, such as alterations in the gut microbiota. Changes in the gut microbiota can affect the balance of Th1/Th2 and Treg/Th17. Tolerogenic dendritic cells can promote the differentiation of T cells into regulatory T cells. Regulatory T cells can not only directly protect against asthma but also influence the balance of Th1/Th2, which is crucial in the onset and progression of asthma.

It’s also unknown if there are any confounding variables besides H. pylori that affect this correlation. Large-scale cohort studies are needed to determine whether the effect of H. pylori on allergic disease is through mediating variables. Further fundamental experimental investigations will be required in the future to investigate and assess these problems and to develop effective strategies for the prevention and treatment of allergic diseases.
